# AMPKα1 Deficiency in Astrocytes from a Rat Model of ALS Is Associated with an Altered Metabolic Resilience

**DOI:** 10.3390/biom13081183

**Published:** 2023-07-28

**Authors:** Inês Belo do Nascimento, Gamze Ates, Nathalie Desmet, Pauline Beckers, Ann Massie, Emmanuel Hermans

**Affiliations:** 1 Institute of Neuroscience, Université Catholique de Louvain, 1200 Brussels, Belgium; ines.belo@uclouvain.be (I.B.d.N.); nathalie.desmet@uclouvain.be (N.D.); pauline.beckers@uclouvain.be (P.B.); 2 Center for Neurosciences, Neuro-Aging & Viro-Immunotherapy, Vrije Universiteit Brussel (VUB), 1090 Brussels, Belgium; gamze.ates@vub.be (G.A.); ann.massie@vub.be (A.M.)

**Keywords:** AMPK, metabolic stress, glutamate transport, ATP

## Abstract

Alterations in the activity of the regulator of cell metabolism AMP-activated protein kinase (AMPK) have been reported in motor neurons from patients and animal models of amyotrophic lateral sclerosis (ALS). Considering the key role played by astrocytes in modulating energy metabolism in the nervous system and their compromised support towards neurons in ALS, we examined whether a putative alteration in AMPK expression/activity impacted astrocytic functions such as their metabolic plasticity and glutamate handling capacity. We found a reduced expression of AMPK mRNA in primary cultures of astrocytes derived from transgenic rats carrying an ALS-associated mutated superoxide dismutase (hSOD1^G93A^). The activation of AMPK after glucose deprivation was reduced in hSOD1^G93A^ astrocytes compared to non-transgenic. This was accompanied by a lower increase in ATP levels and increased vulnerability to this insult, although the ATP production rate did not differ between the two cell types. Furthermore, soliciting the activity of glutamate transporters was found to induce similar AMPK activity in these cells. However, manipulation of AMPK activity did not influence glutamate transport. Together, these results suggest that the altered AMPK responsiveness in ALS might be context dependent and may compromise the metabolic adaptation of astrocytes in response to specific cellular stress.

## 1. Introduction

Characterized by the selective and progressive degeneration of both upper and lower motor neurons, amyotrophic lateral sclerosis (ALS) is the most common motor neuron disease in adults. Muscle denervation leads to progressive weakness, atrophy, and paralysis, which inevitably results in respiratory failure and death. Several pathogenic mechanisms have been elucidated, including excitotoxicity, neuroinflammation, mitochondrial dysfunction and oxidative stress.

Importantly, a profound dysregulation of energy metabolism is observed in both patients and animal models of ALS and is considered a hallmark of the disease [1,2]. Indeed, severe weight loss has been reported and is frequently attributed to decreased food intake and malnutrition. Conversely, high resting energy expenditure, correlating with a systemic hypermetabolism has also been described in ALS patients and animal models [3,4], even though the underlying mechanisms remain unclear. Notably, the metabolic alterations occurring in ALS are suggested to be cell-specific and have been identified in several tissues, particularly in the skeletal muscle and in the central nervous system (CNS). In the brain, alterations in glucose metabolism have been evidenced in the motor cortex of patients [5], as well as decreased levels of ATP [6], suggesting an impaired production of energy. Recently, a hypo-oxidative and hyper-glycolytic metabolic profile has been reported in induced pluripotent stem cells (iPSC)-derived neurons from ALS patients, underlying severe metabolic defects, and resulting in increased vulnerability [7]. Moreover, an increased expression and/or activity of the key cellular energy sensor adenosine monophosphate-activated protein kinase (AMPK) has been described in the spinal cord of both patients and animal models of ALS [8,9,10]. Enhanced activity of this enzyme was also reported in cultured motor neurons expressing ALS-associated mutated forms of the superoxide dismutase 1 (SOD1) [8]. AMPK is a heterotrimeric protein comprising a catalytic alpha (α) subunit and two regulatory subunits, beta (β) and gamma (γ) [11,12]. This enzyme responds to alterations in the energy status of the cell by detecting variations in the adenine nucleotide ratio (AMP/ATP) [13,14,15]. Once activated, AMPK regulates a plethora of cellular pathways, aiming at increasing ATP production and reducing ATP consumption to preserve or restore energy homeostasis [16,17]. A dysregulation of AMPK signaling, particularly highlighted by a hyperactivation of the kinase, has been reported in ALS as well as in other neurodegenerative disorders (reviewed in [18]); however, it remains debated as to whether it is beneficial or detrimental, or if it merely reflects excessive cellular stress.

In the context of ALS, studies on the putative changes in CNS metabolism either appear exclusively focused on motor neurons or fail to address the exact cell-type that is affected. The neurocentric view of ALS has been challenged in recent decades by a strong body of research pointing out at glial cells as main contributors to the progression of the disease [19,20,21]. In particular, astrocytes provide essential trophic, metabolic, and protective support to neurons, and a dysregulation of astrocytic functions has been shown to contribute to several pathogenic mechanisms occurring in ALS [22,23]. For instance, a reduced expression/activity of glial glutamate transporters, as well as an elevated extracellular concentration of glutamate, have been reported in the CNS of both patients and animal models of ALS, contributing to excitotoxic damages to neurons [24,25,26,27,28,29]. Importantly, the metabolic crosstalk between motor neurons and astrocytes also appears disrupted. For instance, decreased expression of the monocarboxylate transporters 1 and 4 (MCT1/4) has been evidenced in astrocytes derived from ALS mouse models, resulting in an impaired lactate shuttling function [30,31]. Moreover, reduced expression of glycogen mobilization enzymes has been reported in iPSC-derived astrocytes from ALS patients as well, resulting in an altered capacity to provide metabolic support to neurons [32,33]. In line with this, aberrant glycogen accumulation has been linked to the neurotoxic phenotype of activated astrocytes in ALS [34]. Furthermore, recent studies have also documented alterations in glucose catabolism in gliosomes (subcellular fractions of astrocytic processes) prepared from the spinal cord of mutant SOD1 mice at symptomatic stages [35,36]. Despite the documented contribution of astrocytes in the regulation of cell metabolism in the CNS and their dysregulation in the course of ALS, the importance of astrocytic AMPK in this context has remained so far barely addressed.

Based on previous studies highlighting an altered control of CNS metabolism in ALS, we herein characterized the expression of AMPKα in primary cultures of astrocytes derived from non-transgenic (Ntg) and transgenic rats carrying an ALS-associated mutated form of the superoxide dismutase 1 gene (hSOD1^G93A^). Our previous work suggested that AMPK regulates the metabolic adaptation to glucose deprivation, as an experimental model of metabolic stress, in an astrocyte-like cell line [37]. Therefore, we hypothesized that a deficiency in AMPK expression or function in astrocytes derived from the transgenic rat model of ALS might alter the astrocyte response to stress and impact on their metabolic plasticity, contributing to the loss of physiological support of nearby neurons.

## 2. Materials and Methods

### 2.1. Animals and Ethics Statement

Experiments were conducted on primary cell cultures derived from Sprague Dawley rat pups in strict accordance with the European Union directive of 22 September 2010 (2010/63/EU) and with the agreement of the Belgian Ministry of Agriculture (code number LA 1230618). The Ethical committee of the Université catholique de Louvain for animal experiments specifically approved this study, which received the agreement number 2018/UCL/MD/031. Animals were housed in cages in controlled light/dark cycles, temperature, and humidity. Every effort was made to minimize suffering during manipulations.

### 2.2. Primary Cultures of Rat Cortical Astrocytes

Primary cultures of astrocytes were generated from rat pups carrying the hSOD1^G93A^ transgene (kindly provided by Dr. R. Pochet; Université Libre de Bruxelles, Belgium). The rat pups were genotyped after birth as previously described [38], and non-transgenic (Ntg) littermates were used as controls. Cortices from newborn rats (post-natal day 1–2) were mechanically dissociated in a standard phosphate buffered saline (PBS) supplemented with glucose 0.2%. Astrocytes were isolated from the other cells through a 30 and 60% Percoll™/RediGrad™ gradient (GE Healthcare, Chicago, IL, USA), and were seeded in 75 cm^2^ culture flasks for a 14-day period of proliferation in Dulbecco’s Modified Eagle’s Medium (DMEM, 21885, ThermoFisher Scientific, Waltham, MA, USA) supplemented with 10% fetal bovine serum (FBS, VWR, Radnor, PA, USA), 100 µg/mL penicillin–streptomycin (ThermoFisher Scientific), 50 µg/mL L-proline (ThermoFisher Scientific), and 2.5 µg/mL amphotericin B (ThermoFisher Scientific) under a humidified atmosphere (5% CO_2_ at 37 °C). The medium was renewed after 7 days. On day 14, cells were collected (trypsin-EDTA, ThermoFisher Scientific) and seeded into multi-well plates or 60 mm culture dishes at the appropriate cell density depending on the type of the experiment. Two days later, the FBS concentration in the culture medium was reduced to 3% during medium renewal to induce astrocyte maturation. All experiments were conducted 7 days later. A last medium renewal was carried out 24 h preceding the day of the experiments.

### 2.3. Glucose Deprivation and Glutamate Exposure Protocols

For the glucose deprivation experiments, standard culture medium containing 5 mM of glucose (DMEM 21885, ThermoFisher Scientific) was replaced with low (0.5 mM) or glucose-free (0 mM) media (DMEM 11966, ThermoFisher Scientific) for 3 h.

For the glutamate exposure experiments, the glucose-containing medium (DMEM 21885) was renewed 3 h before the addition of l-glutamate (250 µM, for 30 min).

### 2.4. Total RNA Extraction and Real-Time Quantitative PCR (RT-qPCR)

For RT-qPCR experiments, astrocytes were seeded in 6-well plates at a density of 200,000 cells/well. The TriPure isolation reagent (Sigma-Aldrich, Saint Louis, MO, USA) was used to extract total RNA from cells, and RNA purity was confirmed by assessing the ratio of absorbance at 260 nm to that at 280 nm. The reverse transcription was then performed using the iScript cDNA synthesis kit (Bio-Rad Laboratories, Hercules, CA, USA) according to the manufacturer’s instructions. Real-time PCR amplifications were carried out as previously described [37]. The following primers were used in the RT-qPCR reactions: *Prkaa1*: 5′-TTAAACCCACAGAAATCCAAACAC-3′ (forward), 5′- CTTCGCACACGCAAATAATAGG-3′ (reverse); *Prkaa2*: 5′-GTGGTGTTATCCTGTATGCCCTTCT-3′ (forward), 5′-CTGTTTAAACCATTCATGCTCTCGT-3′ (reverse); *Gapdh:* 5′-GTCTCCTGTGACTTCAACAG-3′ (forward), 5′-AGTTGTCATTGAGAGCAATGC-3′ (reverse). Plasmid constructs containing the *Prkaa1* or *Prkaa2* rat sequences were used as reference for the absolute quantification of the mRNA levels of the catalytic subunit isoforms α_1_ and α_2_ of AMPK, respectively. The results were expressed as copy number per 10 ng of cDNA.

### 2.5. Western Blot

Cells were seeded into 60 mm cultures dishes at a density of 500,000 cells/dish and Western blot experiments were performed as previously described [37]. Guided by the molecular weight of the protein of interest, the membranes were cut horizontally into several pieces. These pieces were then incubated overnight with the appropriate antibodies recognizing the following proteins: phospho-ACC (1:2000; #3661S, Cell Signaling, Danvers, MA, USA), ACC (1:1000; #3662S, Cell Signaling) and eEF2 (1:1000; PA5-17794, ThermoFisher Scientific). The following day, membranes were washed with TBS-T and were incubated for 1 h at room temperature with the respective peroxidase-conjugated secondary antibodies (Jackson ImmunoResearch, Cambridgeshire, UK). Clarity enhanced chemiluminescence reagent (Bio-Rad Laboratories) was used to detect immunoreactivity, and the densitometry of bands was quantified using ImageJ software (version 1.46r, Wayen Rasband, US National Institutes of Health, Bethesda, MD, USA).

### 2.6. ATP Assay

Cells were seeded into opaque-walled 96-well plates at a density of 10,000 cells/well and a luciferase-based method was used to measure total ATP levels in astrocytes (CellTiter-Glo^®^ 2.0, Promega, Madison, WI, USA), following the manufacturer’s instructions. Relative luminescence units (RLU) were measured using the Victor X-3 Multilabel Plate Reader (Perkin Elmer, Waltham, MA, USA). When comparing basal ATP levels between Ntg and hSOD1^G93A^ astrocytes, results were normalized to protein concentration in sister-wells, avoiding bias of putative differences in cell proliferation. For the glucose deprivation experiments, absorbance values in glucose-deprived conditions were converted as the percentage of the respective control condition (5 mM glucose) and expressed as the difference with the latter.

### 2.7. MTT Assay

Astrocytes were seeded into 96-well plates at a density of 10,000 cells/well. To assess the cell metabolic activity, cells were incubated for 2 h at 37 °C with 100 µL of 0.5 mg/mL 3- (4,5-dimethyl-2-thiazolyl)-2,5-diphenyl-2H-tetrazolium bromide (MTT; Sigma-Aldrich) diluted in the corresponding culture medium. After discarding the supernatant, the reaction was stopped by a 30 min incubation under gentle mixture with a solution of isopropanol/HCl 0.04 N. The Victor X-3 Multilabel Plate Reader was used to measure the absorbance. To avoid bias of putative differences in cell proliferation between Ntg and hSOD1^G93A^ astrocytes, the absorbance values in glucose-deprived conditions were converted as the percentage of the respective control condition (5 mM glucose) and expressed as the difference with the latter.

### 2.8. XF Real Time ATP Rate Assay

Astrocytes were seeded at a density of 5000 cells/well in XF 96 Cell Culture Microplates (Agilent Technologies, Santa Clara, CA, USA). The second day after seeding, FBS was reduced to 3% to induce astrocyte maturation and experiments were carried out 7 days later. On the day of the experiment, after 2 h of exposure to glucose deprivation, cells were washed twice and incubated for 1 h with a DMEM-based XF medium (Agilent Technologies) containing 1 mM sodium pyruvate, 2 mM glutamine, and 10 or 0 mM glucose, in a non-CO_2_ incubator at 37 °C. The Seahorse XFe96 flux analyzer (Agilent Technologies) was used to measure the oxygen consumption rate and the extracellular acidification rate, using the default protocol of the XF Real-Time ATP Rate Assay. Data were normalized by automatic cell counting using the Cytation 1 (Agilent Technologies) after a 30 min incubation in the dark with 4 µM of Hoechst 33,342 (Sigma-Aldrich). Data quality control and initial analyses were performed using the Seahorse Analytics software (Agilent Technologies).

### 2.9. d-[^3^H]-Aspartate Uptake

Cells were seeded into 24-well plates at a density of 30,000 cells/well and placed at the surface of a 37 °C water bath. The activity of glutamate transporters was evaluated by uptake assays using a tracer concentration of 50 nM of radiolabeled d-aspartate (d-[^3^H]-aspartate, specific activity of 12.2 Ci/mmol, Perkin Elmer), as previously described [37]. The use of a single tracing concentration of the substrate, below the known Km value of the transporters, allows for direct evidence of any changes in the Km of Vmax value governing the uptake. Results were expressed as pmol of d-[^3^H]-aspartate transported per min per mg of protein.

### 2.10. Statistical Analyses

Data were obtained from at least three biological replicates (independent experiments conducted on different primary cultures of astrocytes) and were expressed as means with the standard error of the mean (SEM). When mentioned in the figure caption, technical replicates within each experiment were performed. When comparing two datasets, differences between groups were evaluated using paired Student’s *t*-tests, while two-way ANOVA followed by a Bonferroni’s multiple comparisons test were used in two-factor analyses. All statistical analyses were performed on GraphPad Prism Software (version 5.03, San Diego, CA, USA), where a value of *p* < 0.05 was considered as significant.

## 3. Results

### 3.1. AMPK Expression Is Altered in hSOD1^G93A^ Primary Cultures of Astrocytes

**Figure 1:**
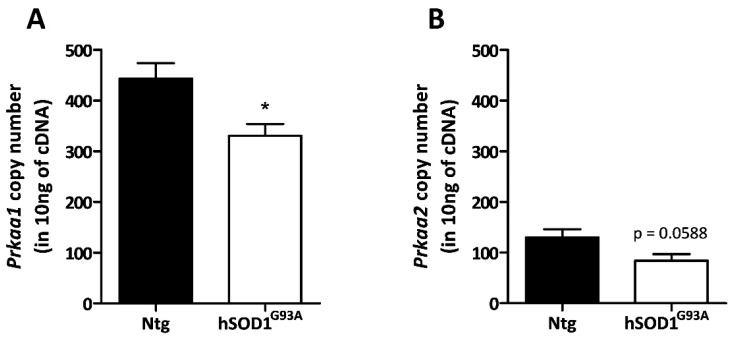
AMPKα mRNA levels in Ntg and hSOD1^G93A^ astrocytes. The mRNA levels of AMPKα_1_ (**A**) and AMPKα_2_ (**B**) isoforms were quantified through RT-qPCR. Plasmid constructs carrying either the *Prkaa1* or *Prkaa2* coding sequences (encoding for AMPKα_1_ and AMPKα_2_, respectively) were used and standards for absolute quantification. Data shown represent the mean ± SEM from five biological replicates. Statistical analyses were performed by Student’s *t* test (* *p* < 0.05).

The mRNAs encoding for both isoforms of the catalytic α subunit of AMPK (α_1_ and α_2_, respectively encoded by *Prkaa1* and *Prkaa2* genes) were quantified by RT-qPCR in primary cultures of astrocytes. These glial cells were grown from non-transgenic (Ntg) or transgenic rats overexpressing the hSOD1^G93A^ transgene. Plasmid constructs carrying either the *Prkaa1* or the *Prkaa2* coding sequences were used as reference for absolute quantification. As shown in Figure 1, primary cultures of astrocytes are characterized by a predominant expression of the α_1_ isoform in comparison to the α_2_ isoform (>3-fold difference). Moreover, the expression level of the catalytic α subunits of AMPK was found to be lower in hSOD1^G93A^ astrocytes compared to their Ntg counterparts (respectively, 330 and 443 copies/10 ng cDNA for α_1_, *p* < 0.05; 83 and 129 copies/10 ng cDNA for α_2_, *p* = 0.06). Given the crucial role of AMPK in the maintenance of energy homeostasis, these observations suggest that hSOD1^G93A^ astrocytes might be less equipped to face cellular insults.

### 3.2. Pharmacological Activators Induce AMPK Activity in Both Ntg and hSOD1^G93A^ Astrocytes

**Figure 2:**
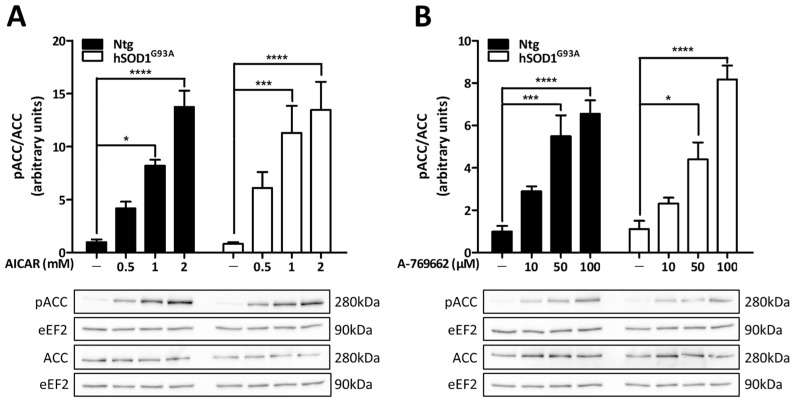
Pharmacological activation of AMPK in Ntg and hSOD1^G93A^ astrocytes. Primary cultures of astrocytes derived from Ntg or hSOD1^G93A^ rats were exposed for 3 h to increasing concentrations of two pharmacological activators of AMPK: AICAR (**A**) and A-769662 (**B**). AMPK activity was assessed by immunoblot analyses of the phosphorylation levels of ACC, a downstream target of AMPK. Phosphorylated and total levels of ACC were detected on different membranes and their expression was normalized to that of eEF2. Blots shown are representative of at least four independent experiments and histograms represent the means ± SEM. Statistical analyses were performed by two-way ANOVA followed by Bonferroni’s multiple comparison test (* *p* < 0.05, *** *p* < 0.001, **** *p* < 0.0001).

To test whether AMPK could be activated in cultured astrocytes, Ntg and hSOD1^G93A^ cells were exposed for 3 h to increasing concentrations of two distinct AMPK pharmacological activators: 5-aminoimidazole-4-carboxamide-1-β-D-ribofuranoside (AICAR; Toronto Research Chemicals; 0.5, 1 or 2 mM dissolved in the culture medium) and compound A-769662 (TOCRIS; 10, 50 or 100 µM pre-dissolved in DMSO—final maximal concentration of DMSO in the culture medium of 0.1%). Immunoblot analysis of the phosphorylation of acetyl-CoA carboxylase (ACC) at serine 79 residue—a well-characterized phosphorylation site for AMPK—was used as an indicator of AMPK activity. Basal levels of ACC phosphorylation were similar between Ntg and hSOD1^G93A^ astrocytes. In both AICAR- (Figure 2A) and A-769662- (Figure 2B) treated cells, the pACC/ACC ratio was increased in a concentration-dependent manner, but no significant difference was observed between Ntg and hSOD1^G93A^ cells. Thus, despite the altered mRNA levels of the AMPKα subunits in hSOD1^G93A^ cells, the activity of AMPK was stimulated to the same extent as in Ntg astrocytes by pharmacological activators.

### 3.3. Altered AMPK Activity in hSOD1^G93A^ Astrocytes

**Figure 3:**
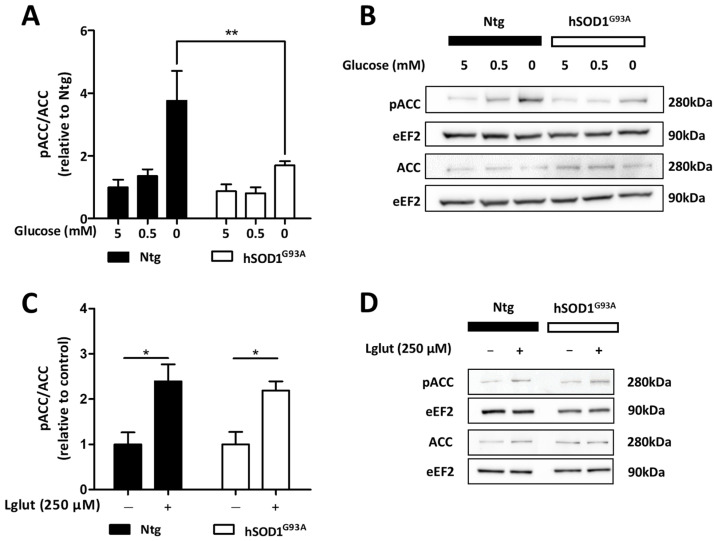
AMPK activity in Ntg and hSOD1^G93A^ astrocytes in response to metabolic challenges. Ntg and hSOD1^G93A^ astrocytes were exposed to partial (0.5 mM) or total glucose-deprived conditions (**A**,**B**) or to a high concentration of glutamate (Lglut; 250 µM) for 3 h (**C**,**D**) in order to induce cellular stress. The activity of AMPK was assessed by immunoblot analyses of the pACC/ACC ratio. Phosphorylated and total ACC were detected on different membranes and their expression was normalized to that of eEF2. (**A**,**C**) Histograms show means ± SEM and blots shown are representative of eight (**B**) or four (**D**) independent experiments. Statistical analyses were performed via two-way ANOVA followed by Bonferroni’s multiple comparison test (* *p* < 0.05, ** *p* < 0.01).

AMPK responds to alterations in the cellular energy balance, and metabolic stress elicited by energetic substrate deprivation has been shown to promote AMPK activation in several cell types. Thus, we sought to determine whether glucose deprivation stimulated the activity of AMPK in astrocytes. Primary cultures of Ntg and hSOD1^G93A^ astrocytes were challenged with low (0.5 mM) and glucose-free media for 3 h. When the glucose concentration was decreased to 0.5 mM, no significant increase in AMPK activity could be detected in either Ntg or hSOD1^G93A^ astrocytes (Figure 3A,B). However, 3 h of total (0 mM) glucose deprivation increased by 3.8-fold the pACC/ACC ratio in Ntg astrocytes, thus reflecting a significant increase in the activity of AMPK in response to glucose deprivation. In hSOD1^G93A^ astrocytes, the increase in the pACC/ACC ratio elicited by glucose deprivation was significantly lower compared to their Ntg counterparts (1.9-fold). These observations suggest that the responsiveness of AMPK to glucose deprivation is altered in hSOD1^G93A^ astrocytes compared to Ntg cells and might compromise their ability to face metabolic stress.

Repeated exposure to high concentrations of glutamate during synaptic activity is likely to challenge the metabolic activity of astrocytes. Indeed, these cells ensure efficient uptake of extracellular glutamate at the expense of Na^+^ exchange, which, in turn, requires ATP to restore proper ionic membrane gradients. Thus, we sought to determine whether the exposure to a high concentration of glutamate (250 µM for 30 min) enhanced the activity of AMPK in astrocytes. Analysis of immunoblots probing for pACC and ACC shown in Figure 3C,D revealed that such exposure to glutamate induces an increase in AMPK activity in Ntg astrocytes, as evidenced by a 130% increase in the pACC/ACC ratio. Glutamate exposure also caused an increase in the activity of AMPK in hSOD1^G93A^ astrocytes, which was found to be similar to that observed in Ntg cells. These data suggest that the altered AMPK response previously observed after glucose deprivation in ALS-derived astrocytes might be specific for this severe metabolic challenge and could therefore be stress-type-dependent.

### 3.4. Metabolic Adaptation of Ntg and hSOD1^G93A^ Astrocytes during Glucose Deprivation

**Figure 4:**
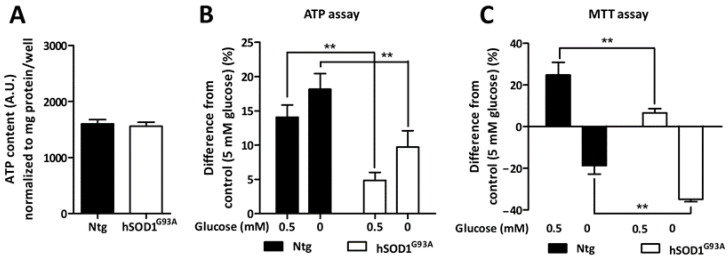
Ntg and hSOD1^G93A^ astrocytes response to glucose-deprivation. (**A**,**B**) ATP content was assessed using a luciferase-based assay. Histograms show means ± SEM from four independent experiments, in which each experimental condition was tested in quadruplicates. (**A**) Basal ATP levels were normalized to that of protein content in sister-wells. (**B**) The results obtained in glucose-deprived conditions are expressed as the difference from the respective control condition (5 mM of glucose). (**C**) Metabolic activity was evaluated by MTT colorimetric assay. Histograms show means ± SEM from eight independent experiments. Statistical analyses were performed either by Student’s *t* test (**A**) or by two-way ANOVA followed by Bonferroni’s multiple comparison test (** *p* < 0.01) (**B**,**C**).

As the responsiveness of AMPK to glucose deprivation was found to be altered in cells derived from the ALS rat model, we sought to further characterize the astrocytic response to this metabolic stress. For that purpose, the intracellular ATP content was measured in cultured Ntg and hSOD1^G93A^ cells exposed to low (0.5 mM) and glucose-free media for 3 h. In the absence of such stress, basal ATP levels were shown to be similar between Ntg and hSOD1^G93A^ astrocytes (Figure 4A). In response to both mild and severe glucose deprivation, Ntg astrocytes increased their ATP content by 14 and 18%, respectively (Figure 4B). Astrocytes derived from the hSOD1^G93A^ rodents also increased their ATP levels in both glucose-deprived conditions (4.8 and 9.7% when exposed to 0.5 mM or 0 mM of glucose, respectively). However, this increase was significantly lower compared to what was observed in Ntg cells (Figure 4B).

The metabolic activity of Ntg and hSOD1^G93A^ cells was then measured using the MTT assay (Figure 4C). Ntg astrocytes exposed to a mild (0.5 mM) glucose deprivation showed a 24.8% increase in their metabolic activity. In line with the previous results, the response of hSOD1^G93A^ astrocytes to a mild (0.5 mM) glucose deprivation was less pronounced compared to Ntg cells (6.6% increase in metabolic activity for hSOD1^G93A^ astrocytes). Contrariwise, severe glucose deprivation caused an 18.7% decrease in the activity revealed by the MTT test in Ntg astrocytes, suggesting that either the viability and/or the metabolism of the cells might be compromised. Strikingly, hSOD1^G93A^ astrocytes showed a greater decline in the metabolic activity measured with the MTT assay compared to Ntg astrocytes (34.8% for hSOD1^G93A^ astrocytes). Together with the results obtained when measuring the alterations in ATP levels, these data suggest that cultured astrocytes derived from the hSOD1^G93A^ rodent model of ALS show an altered response to metabolic stress compared to their Ntg counterparts.

**Figure 5:**
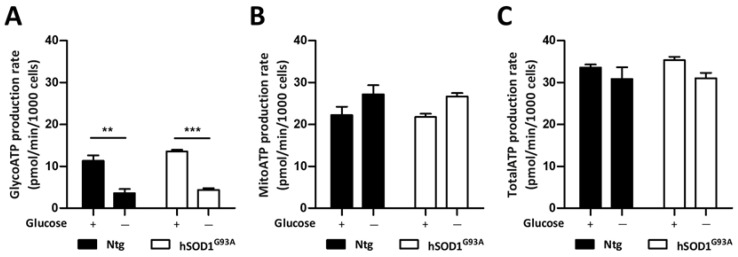
ATP production rate of Ntg and hSOD1^G93A^ cells exposed to glucose deprivation. The oxygen consumption rate and the extracellular acidification rate of Ntg and hSOD1^G93A^ astrocytes were measured using the Seahorse XFe96 metabolic flux analyzer. An ATP rate assay was carried out after cells were exposed to glucose-deprived conditions for 3 h. Histograms show means ± SEM obtained from three biological replicates, and represent glycolytic (**A**), mitochondrial (**B**), and total (**C**) ATP production rates. Statistical analyses were performed by two-way ANOVA followed by Bonferroni’s multiple comparison test (** *p* < 0.01, *** *p* < 0.001).

Further analyses of the metabolic activities of astrocytes were obtained through the Seahorse XF Real-Time ATP Rate Assay (Figure 5). No difference was found between the basal metabolic activities of Ntg and hSOD1^G93A^ astrocytes. When cells were exposed to glucose deprivation a clear reduction in the glycolytic ATP production rate was observed in both cell types (Figure 5A). This was accompanied by a slight increase in the mitochondrial ATP production rate (Figure 5B), resulting in a barely modified total ATP production rate in both Ntg and hSOD1^G93A^ cells (Figure 5C).

Together, these results indicate that despite the increased ATP levels observed after glucose deprivation, astrocytes maintain stable levels of ATP production. It is likely that a concomitant down-regulation of ATP-consuming processes is responsible for the increase in total ATP content. Additionally, these results suggest that both Ntg and hSOD1^G93A^ astrocytes are resilient to metabolic stress.

### 3.5. Regulation of Glutamate Uptake Does Not Rely on the Activity of AMPK in Both Ntg and hSOD1^G93A^ Astrocytes

**Figure 6:**
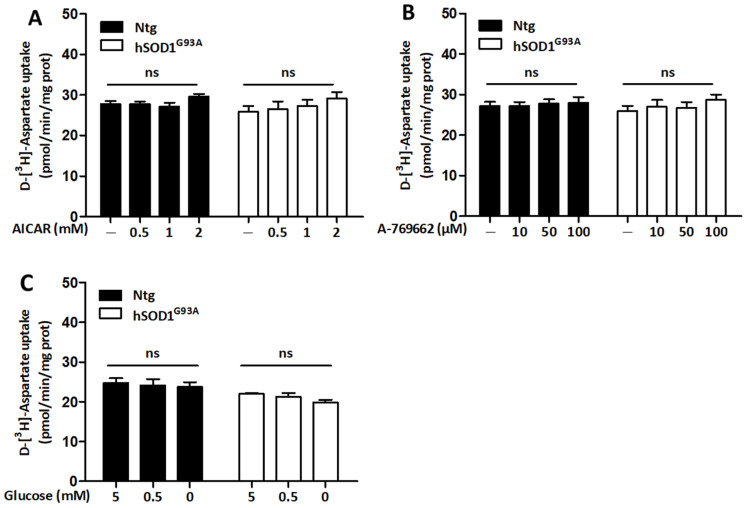
Effect of AMPK activation on the activity of glutamate transporters. Primary cultures of Ntg and hSOD1^G93A^ astrocytes were treated with increasing concentrations of the pharmacological activators of AMPK AICAR (0.5, 1 and 2 mM) (**A**) and A-769662 (10, 50 and 100 µM) (**B**) for 3 h. As an indirect way of triggering AMPK activity, cells were exposed to 3 h of partial (0.5 mM) or total glucose deprivation (**C**). A tracing concentration (50 nM) of radiolabeled d-aspartate was used to measure the activity of glutamate transporters. Histograms show means ± SEM of data obtained from three (**A**,**B**) or five (**C**) independent experiments performed in quadruplicates. Statistical analyses were carried out via two-way ANOVA followed by Bonferroni’s multiple comparison test (ns: non-significant).

As an energy consuming process, we hypothesized that glutamate uptake in astrocytes might be downregulated by AMPK. The activity of glutamate transporters was therefore measured in astrocytes exposed to the pharmacological activators of AMPK (Figure 6A,B). A tracing concentration of radiolabeled d-aspartate (50 nM) was used to assess the activity of glutamate transporters. The basal activity of glutamate transporters was found to be similar in Ntg and hSOD1^G93A^ astrocytes. Activation of AMPK with AICAR (Figure 6A) and the A-769662 compound (Figure 6B) was without influence on the activity of glutamate transporters, in both cell types. The activity of glutamate transporters in Ntg and hSOD1^G93A^ astrocytes was also measured when cells were exposed to partial (0.5 mM) and total glucose deprivation, an indirect approach to trigger AMPK activity. In these conditions, no significant alteration in d-aspartate uptake was detected in both Ntg and hSOD1^G93A^ astrocytes (Figure 6C). Together, these observations suggest that the activity of glutamate transporters is not influenced by the activity of AMPK and does not seem to be regulated in response to metabolic stress conditions.

## 4. Discussion

With a rather limited capacity of energy storage, neurons highly depend on the continuous supply of energetic substrates, a process that is tightly controlled by nearby astrocytes. These glial cells are indeed anatomically and functionally positioned to couple the CNS microvasculature to neuronal energy demands and act as mandatory partners in the regulation of brain metabolism. Emphasizing their strong metabolic partnership, astrocytes and neurons distinctly express specific transporters for energetic substrates, as well as specific enzymes supporting their complementary metabolic profiles [39,40]. Metabolic alterations in the CNS that affect the function or viability of neurons have been documented in several neurodegenerative disorders, and several studies have indeed examined the importance of metabolic dysfunction in neurons as a key pathogenic mechanism. At variance, and despite their documented implication in the progression of several neurodegenerative diseases, astrocytes and their metabolic adaptation have so far received little consideration. In particular, the role and regulation of AMPK, which operates as an essential metabolic gauge in the majority of eukaryotic cells, have not been studied in astrocytes in models of neurodegenerative disorders. In the present study, we aimed at characterizing the expression and activity of AMPK in primary cultures of astrocytes prepared from newborn rat pups. It is important to note that despite the evident limitations that stem from the use of animals of young age in the context of age-associated disorders, these cells constitute a relevant in vitro model to detect very early/constitutive alterations that might compromise the proper function of glial cells in the long term. As such, it is a cell model that has been extensively used to study the implication of astrocytes in neurodegenerative disorders, and notably ALS. Further studies on adult astrocytes prepared from animals at symptomatic stages of ALS would add significant value to our observations.

The seminal work of Turnley et al. in 1999, examining the expression of the diverse AMPK isoforms/subunits in vivo, had concluded that AMPK is only expressed in reactive astrocytes [41]. More recently, the detection of AMPK in these glial cells has been documented in several in vitro studies conducted on primary cultures [42,43]. Commonly characterized by high expression of GFAP, astrocytes in culture commonly adopt an activated phenotype, which may explain these observations [44]. Nevertheless, studies conducted in transgenic animals demonstrated that the suppression of AMPK in astrocytes affects neuronal survival [45]. We have previously studied the expression of AMPKα isoforms in an astrocyte-like cell model (C6 glioma cells), where AMPKα_1_ was identified as the predominantly expressed catalytic subunit [37]. Consistent with these findings, we herein found that the α_1_ isoform is also predominantly expressed in primary cultures of astrocytes. Importantly, our data evidenced a difference in the expression of AMPKα subunits in astrocytes cultures derived from Ntg rats or from rats expressing an ALS-associated mutated form of the SOD1 enzyme. To the best of our knowledge, there are no reports on a link between SOD1 and the regulation of AMPK gene expression. In fact, the regulation of AMPK genes has been poorly characterized so far [46]. Nonetheless, the lower mRNA levels of both AMPKα_1_ and AMPKα_2_ observed in cells derived from hSOD1^G93A^ rats suggests that these astrocytes are less equipped to face stress conditions, which may be of relevance in this animal model of ALS.

Despite the difference in expression of the AMPK catalytic subunits, the activity of AMPK measured in the absence of metabolic stress was found to be similar in Ntg and hSOD1^G93A^ cells. Consistently, in standard culture conditions, similar ATP content was measured in both cell types, as well as comparable basal ATP production rates. It is worth noting that cultured cells are commonly maintained in optimal conditions, particularly regarding nutriment availability, that certainly do not recapitulate the in vivo microenvironment or the context of the modeled diseases. In the C6 cell model, alteration in glucose availability was indeed shown to trigger metabolic adaptation through an AMPK-dependent process [37]. Moreover, severe glucose deprivation in this cell line was found to alter their metabolic activity, as evidenced by a decrease in the MTT reduction capacity. Similarly, primary cultures of astrocytes were herein exposed to these metabolic stress conditions in order to appreciate their metabolic plasticity. Consistent with the observations in C6 cells, severe glucose-deprivation caused a decrease in the MTT assay readout in primary cultures of astrocytes. As expected, this was accompanied by a remarkable activation of AMPK. Such response was found to be altered in hSOD1^G93A^ astrocytes, where notably, the AMPK responsiveness to stress was considerably decreased. It is important to highlight that the present investigation has been carried out in primary cultures of astrocytes prepared from the cortex of rat pups. As such, it is noteworthy that substantial differences have also been highlighted in primary cultures of astrocytes prepared from distinct regions of the CNS in the context of ALS [47,48]. Hence, the alterations reported herein are possibly specific to cortical astrocytes and should not be generalized to regionally distinct astrocytes.

Consistent with previous reports [49], astrocytes show an increase in their ATP content in response to glucose deprivation. In astrocytes derived from the ALS rat model, we found that ATP levels were differentially modified, as their increase in the absence of glucose appeared less pronounced compared to Ntg cells. Strikingly, the Seahorse XF Real-Time ATP Rate Assay revealed a similar adaptation of ATP-producing pathways in both cell types exposed to glucose deprivation, which consisted of a switch from glycolytic to mitochondrial ATP production. Of note, the similarities between the metabolic profiles of hSOD1^G93A^ and Ntg neonatal astrocytes are consistent with recent reports from Ravera and colleagues. Indeed, minor metabolic alterations were evidenced in gliosomes (preparations of glial subcellular processes) obtained from the spinal cord of adult, symptomatic, SOD1^G93A^ mice. These alterations were not found at pre-symptomatic stages as opposed to what could be observed in the neuronal counterpart (synaptosomes) [35,36]. Taken together, our data suggest that the decreased responsiveness of AMPK to glucose deprivation in the cells from the ALS model does not influence their ability to regulate their production of ATP but could more likely alter their capacity to adapt metabolic activities that consume ATP. This is consistent with our previous study, where we proposed a link between a reduced AMPK activity and an altered capacity to regulate energy consumption in astrocyte-like cells [37]. Interestingly, it has recently been proposed that depending on the intensity of the cellular stress, different pools of AMPK complexes might be activated within the cell. These may have specific downstream targets in line with their different subcellular location [50,51]. For instance, Zong et al. have demonstrated that glucose deprivation for 2 h essentially activates lysosomal AMPK pools, mainly leading to anti-anabolic effects such as the inhibition of protein and fatty acid synthesis [50]. Thus, the regulation of catabolism by AMPK might occur later, after prolonged or more severe cellular stress. Such a graded response of AMPK to cellular stresses could explain the lack of effect on the regulation of ATP production reported in our study, as astrocytes were exposed to an acute deprivation of glucose (3 h).

It is worth mentioning that an impaired AMPK activation in response to cellular stress has been already reported, notably in the context of aging. For instance, Reznick et al. documented that AICAR treatment and physical exercise increased AMPK activity in the muscles of young rats, whereas such adaptation was not detected in old rats [52]. Similarly, in the brain, Liu et al. demonstrated that cerebrovascular stroke induced a robust increase in AMPK activity in young mice whereas in old mice, AMPK activity remained unaffected [53]. This alteration in AMPK responsiveness to cellular stress is proposed to impair the cell metabolic regulation, increase oxidative stress, and reduce autophagic clearance [54]. In the context of ALS, a loss of sensitivity of AMPK to stress—particularly in astrocytes—could compromise essential astrocytic functions and impair the astrocytic support to neurons.

One of the key roles played by astrocytes in the nervous system is to preserve glutamate homeostasis. Astrocytes ensure the clearance of glutamate from the synaptic cleft through the activity of dedicated glutamate transporters to terminate neuronal transmission and avoid excessive and deleterious excitation. This function was shown to be impaired in ALS, where excitotoxicity is recognized as one of the hallmarks of this degenerative disease [29]. In cultured astrocytes exposed to l-glutamate, a decrease in energy (ATP) levels has been reported [55] and lead to an increase in glucose uptake [56]. However, even if previous studies have focused on the functional coupling between glutamate uptake and energetic substrate mobilization through the activity of glucose transporters, the exact molecular mechanisms still remain unclear. Thus, we had hypothesized that exposure to high concentrations of glutamate might trigger AMPK activation in astrocytes, and that this could be linked to the energy consumption resulting from the maximal solicitation of glutamate transporters. Consistent with what has been reported in neurons [57], glutamate exposure was herein found to increase AMPK activity in astrocytes. It is noteworthy that a similar increase in the activity of AMPK was observed in ALS-derived astrocytes. These results were unexpected given the diminished response of AMPK observed in hSOD1^G93A^ cells in response to the previous cellular stress tested (i.e., glucose deprivation). Thus, this suggests that the altered responsiveness of AMPK observed in hSOD1^G93A^ cells might be dependent on the nature of the cellular stresses. A better understanding of these different contexts could help decipher new cellular mechanisms that are altered in glial cells in ALS and that compromise their resilience.

As glutamate uptake is indirectly linked to ATP consumption to preserve ionic gradients, we had hypothesized that glutamate transporters might figure among the AMPK targets which become downregulated to preserve ATP. Notably, the reports in the literature on the regulation of glutamate uptake by AMPK are controversial. For instance, while Voss et al. found that AMPK activation with AICAR had no effect on glutamate transport in cultured astrocytes [58], Maixner et al. found that pharmacological activation of AMPK with the same compound increased glutamate uptake in spinal cord slices [59]. It is important to consider that the in vitro conditions—which are often optimized for cultured cell functions—might not mimic the cellular context in stress conditions and might lead to discrepancies between in vitro and in vivo studies. Notwithstanding, consistent with the reports from Voss et al., we found that the pharmacological activation of AMPK with AICAR or A-769662 had no effect on the activity of glutamate transporters in primary cultures of astrocytes. Similarly, glucose-deprivation did not alter glutamate uptake in these cells. Our results suggest that even in unfavorable conditions where AMPK is activated (i.e., stress), glutamate clearance by astrocytes remains unaffected and could even be prioritized as to preserve the support to neuronal activity and viability. It is even tempting to speculate that in astrocytes challenged with stress conditions, AMPK might repress other ATP consuming pathways in order to secure the energy load necessary for glutamate uptake.

In the present study, we have focused on the link between AMPK and glutamate transport. Nevertheless, this enzyme has a multitude of downstream targets and regulates many other cellular pathways which, notably, have been reported to be altered in ALS as well. For instance, in motor neurons, aberrant AMPK signaling has been proposed to be responsible for the metabolic shift from the pentose phosphate pathway towards glycolysis, compromising the cellular redox balance [60]. In astrocytes, elevated glycogen synthesis has recently been highlighted as a major contributor to neurotoxicity [34]. An impairment in AMPK activity in these glial cells could indeed compromise the regulation of glucose storage as glycogen and lead to its accumulation. Moreover, an impairment in autophagy has recently been reported in iPSC-derived astrocytes carrying the hSOD1^G93A^ mutation [61]. Thus, it is tempting to speculate that the altered AMPK signaling in hSOD1^G93A^ astrocytes reported herein could also bear consequences at the level of autophagy regulation. Considering this, further studies are needed in order to clarify which downstream pathways are affected in these cells.

## 5. Conclusions

In summary, we found that AMPK sensitivity to metabolic stress was altered in neonatal astrocytes prepared from the cortex of an ALS rat model. Considering the documented role of AMPK in the majority of mammalian cells, this would make these cells more vulnerable to this specific stress, as indicated by a more limited increase in ATP levels in response to glucose deprivation compared to Ntg cells, as well as a more pronounced decrease in metabolic activity observed in the MTT assay. Importantly, this might bear severe consequences as it may compromise some astrocytic supportive functions towards neurons. The altered AMPK responsiveness was, however, found to be context dependent, as other stimuli did not reveal any difference in AMPK activity between Ntg and hSOD1^G93A^ cells. Further investigations should be conducted in order to better understand how AMPK is regulated in astrocytes in ALS and how it may have an impact on the progression of the disease.

## Data Availability

The data presented in this study are available from the corresponding author on request.
